# Intense Broadband Emission in the Unconventional 3D Hybrid Metal Halide via High‐Pressure Engineering

**DOI:** 10.1002/advs.202306937

**Published:** 2023-12-24

**Authors:** Xuening Sun, Min Wu, Xihan Yu, Qian Li, Guanjun Xiao, Kai Wang, Bo Zou

**Affiliations:** ^1^ State Key Laboratory of Superhard Materials College of Physics Jilin University Changchun 130012 China; ^2^ Shandong Key Laboratory of Optical Communication Science and Technology School of Physics Science and Information Technology Liaocheng University Liaocheng 252000 China

**Keywords:** 3D hybrid metal halide, bandgap narrowing, pressure‐induced emission, self‐trapped states

## Abstract

Developing hybrid metal halides with self‐trapped exciton (STE) emission is a powerful and promising approach to achieve single‐component phosphors for wide‐color‐gamut display and illumination. Nevertheless, it is difficult to generate STEs and broadband emission in the classical and widely used 3D systems, owing to the great structural connectivity of metal‐halogen networks. Here, high pressure is implemented to achieve dual emission and dramatical emission enhancement in 3D metal halide of [Pb_3_Br_4_][O_2_C(CH_2_)_2_CO_2_]. The pressure‐induced new emission is ascribed to the radiation recombination of STEs from the Pb_2_Br_2_O_2_ tetrahedra with the promoted distortion through the isostructural phase transition. Furthermore, the wide range of emission chromaticity can be regulated by controlling the distortion order of different polyhedral units upon compression. This work not only constructs the relationship between structure and optical behavior of [Pb_3_Br_4_][O_2_C(CH_2_)_2_CO_2_], but also provides new strategies for optimizing broadband emission toward potential applications in solid‐state lighting.

## Graphical abstract

Dramatical self‐trapped exciton emission enhancement and the wide range of emission chromaticity regulation are realized in the 3D metal halide [Pb_3_Br_4_][O_2_C(CH_2_)_2_CO_2_] by pressure engineering, which is ascribed to controlling the distortion of different polyhedral units. This study provides a potential pathway for the design of 3D metal halides with broadband and optimized PL properties.

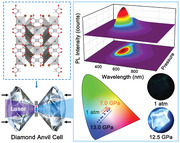

## 1 Introduction

The self‐trapped exciton (STE) in metal halides refers to a class of localized carriers trapped by the lattice deformation potential with strong electron‐phonon coupling effect.^[^ ^1^ ^]^ The unique features of STE endow these materials with large Stokes shift and broadband photoluminescence (PL) emission, providing excellent platforms to develop efficient phosphors for displays, light‐emitting diodes, single‐component white‐light illumination, etc.^[^ ^2^ ^]^ On the basis of the essential requirements of structural confinement for excitonic trapping, STE emission is commonly achieved in low‐dimensional metal halides.^[^ ^3^ ^]^ It is still challenging to generate STEs in the state of the art 3D systems, which exhibit great structural connectivity with wide and multifunctional applications in practice.^[^ ^4^ ^]^

One of the principal methods to develop STE emission in 3D metal halide is decorating the local electronic structure by accurate composition design. Recently, ionic doping is proposed as a novel strategy to promote lattice distortion, generating STEs in 3D metal‐halogen networks and leading to certain excitonic emission from self‐trapped states.^[^ ^5^ ^]^ Nevertheless, the structural stability and optical properties of these materials are influenced inevitably by the permanent defects, which greatly hinder the further photoelectric applications and the subsequent material design with targeted properties. Different from the ionic‐doped systems, the 3D metal halide [Pb_3_Br_4_][O_2_C(CH_2_)_2_CO_2_] is constructed by the interpenetrated succinic acid and bromoplumbate frameworks, which are considerably stable at ambient conditions (1 atm).^[^ ^6^ ^]^ The highly distorted network brings about the rare STE emission in 3D material without ionic doping. Note that there are two photosensitive units of Pb_2_Br_4_O_2_ octahedra and Pb_2_Br_2_O_2_ tetrahedra in [Pb_3_Br_4_][O_2_C(CH_2_)_2_CO_2_], which should result in two sets of self‐trapped states with dual‐band STE emissions and wide color gamut. Whereas, [Pb_3_Br_4_][O_2_C(CH_2_)_2_CO_2_] only exhibits single STE emission peak with a relatively low photoluminescence quantum yield of 1.8% at 1 atm. The emission mechanism of this material is relatively obscure. Hence, it is of critical importance to optimize and accurately modulate the structure and PL property of [Pb_3_Br_4_][O_2_C(CH_2_)_2_CO_2_], getting an in‐depth study of the STE emission mechanism in this unique 3D materials.

As a cleaning method to modify the electronic landscape and crystalline structures without composition variation, pressure has been widely applied to regulate and control the PL emission, bandgap, photocurrent and phase transformation of metal halides.^[^ ^7^ ^]^ Especially, in low‐dimensional metal halides, pressure‐induced polyhedral distortion and vibration confinement can trigger the enhancement and discoloration of the STE emission.^[^ ^8^ ^]^ Analogously, high‐pressure exploration on [Pb_3_Br_4_][O_2_C(CH_2_)_2_CO_2_] is expected to modulate the crystalline structure and STE emission, construct the detailed structure‐property relationships and thus elucidating the underlying emission mechanism.

Here, a systematic high‐pressure study was carried out on [Pb_3_Br_4_][O_2_C(CH_2_)_2_CO_2_]. With increasing pressure, a new emission band was emerged at 8.0 GPa and the PL intensity achieved 130‐fold enhancement at 12.5 GPa. The fluorescence color was also dramatically varied from dull white to bright bluish‐white. Based on first‐principles calculations and in situ high‐pressure experiments, including PL and UV–vis absorption, synchrotron X‐ray diffraction (XRD), Raman and infrared absorption, it is confirmed that the variations of PL spectra should be ascribed to the isostructural phase transition at 7.5 GPa. During the phase transition, the nonluminous Pb_2_Br_2_O_2_ tetrahedra is distorted considerably, promoting the new STE emission from this photoactive component. Meanwhile, both the contraction of lattice and the increase in the distortion degree of Pb_2_Br_2_O_2_ tetrahedra and Pb_2_Br_4_O_2_ octahedra upon compression result in a wide range modulation of fluorescence chromaticity. This study not only reveals the emission mechanism of [Pb_3_Br_4_][O_2_C(CH_2_)_2_CO_2_], but also provides a potential pathway for the design of 3D metal halides with broadband and optimized PL properties.

## 2 Results and Discussion

**Figure 1:**
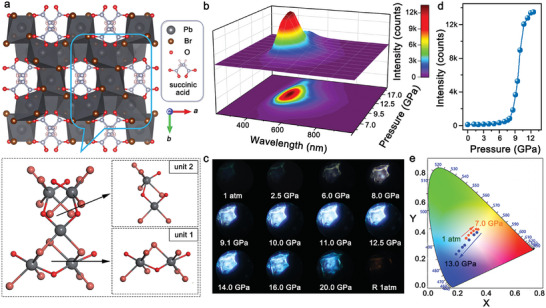
a) Crystalline structure of [Pb_3_Br_4_][O_2_C(CH_2_)_2_CO_2_] viewed perpendicular to the *c* axis (up), and the selected periodic structure (down). The unit 1 is two Pb_2_Br_4_O_2_ octahedra connected along *a*‐axis and unit 2 consists of Pb_2_Br_4_O_2_ octahedra and Pb_2_Br_2_O_2_ tetrahedra connected along *b*‐axis. b) Pressure‐dependent 3D colormap surface with the projection of PL spectra. c) PL micrographs of [Pb_3_Br_4_][O_2_C(CH_2_)_2_CO_2_] crystal upon compression and decompression with the excitation at 355 nm. d) Variations in the PL intensity as a function of pressure. e) Chromaticity coordinates of [Pb_3_Br_4_][O_2_C(CH_2_)_2_CO_2_] with the increasing pressure from 1 atm to 13.0 GPa.

In [Pb_3_Br_4_][O_2_C(CH_2_)_2_CO_2_], the adjacent [PbBr]^+^ chains are vertex‐bridged by the Pb_2_Br_4_ units (**Figure** **1**a). The Pb centers in [PbBr]^+^ chains and Pb_2_Br_4_ pillars occupy different geometries of octahedra and tetrahedra, respectively. Succinic acid, as structure‐directing agent, is connected with Pb^2+^ through covalent bond to support the 3D network and enhance structural stability (Figure S1, Supporting Information). As depicted in Figure 1a, the overall structure of [Pb_3_Br_4_][O_2_C(CH_2_)_2_CO_2_] defines eight‐membered ring channels along the *c*‐axis. For the convenience of observation, a periodic structural unit was chosen for study. This structural unit consists of two Pb_2_Br_4_O_2_ octahedra arranged along *a*‐axis (labeled as unit 1), as well as the connected Pb_2_Br_4_O_2_ octahedra and Pb_2_Br_2_O_2_ tetrahedra along *b* axis (labeled as unit 2). Based on the highly deformable and anharmonic lattice structure, the relatively strong electron‐phonon coupling effect in [Pb_3_Br_4_][O_2_C(CH_2_)_2_CO_2_] contributes to the trapping of photo‐generated free excisions (FEs), giving rise to the subsequent STE emission in this 3D material.^[^ ^6^ ^]^

In order to explore the pressure effects on the STE emission of [Pb_3_Br_4_][O_2_C(CH_2_)_2_CO_2_], in situ high‐pressure PL measurements were carried out up to 24.0 GPa (Figure 1b,c; Figures S2 and S3, Supporting Information). At 1 atm, the PL emission (defined as peak I) of [Pb_3_Br_4_][O_2_C(CH_2_)_2_CO_2_] was centered at ≈505 nm under the photoexcitation of 355 nm. With increasing pressure to 7.0 GPa, the peak I exhibited a ≈50 nm redshift with slightly increased

intensity. The full width at half‐maximum (FWHM) of peak I increased by 27 nm (Figure S4, Supporting Information). Then, a new emission peak (defined as peak II) appeared at 8.0 GPa with a shorter emission wavelength of ≈487 nm (Figure S5, Supporting Information). Upon further compression, the intensity of peak II was significantly enhanced and the PL intensity at 12.5 GPa was ≈130 times stronger than that at 1 atm (Figure 1d; Figure S6, Supporting Information), which is the highest value among all reported 3D metal halides with pressure‐induced emission enhancement properties (Table S1, Supporting Information). Both the peak I and peak II were blue‐shifted with the FWHM contraction between 8.0 and 13.0 GPa, which promoted the transformation of fluorescence (Figure S7, Supporting Information). To quantitively describe the variation of emission color, the Commission Internationale de l´ Eclairage (CIE) chromaticity coordinates and the correlated color temperature (CCT) were also recorded (Figure 1e; Tables S2 and S3, Supporting Information). Between 1 atm and 7.6 GPa, the CIE coordinate was gradually modulated from (0.27, 0.35) to (0.34, 0.39) and the emission color was accordingly changed from “cold” white (8684 K) to pure white (5155 K). Upon further compression to 13.0 GPa, the CIE coordinate changed to (0.19, 0.24), and the emission color transferred obviously to the bright bluish‐white emission with the CCT of 950 000 K. From the structural viewpoint, these PL variations should be ascribed to the pressure‐induced regulation of the electron‐phonon coupling and polyhedral conformation.^[^ ^9^ ^]^ Meanwhile, the redshifts and weakening of peaks above 12.5 GPa should be related to the gradual structural destruction (Figure S3, Supporting Information).^[^ ^10^ ^]^ Additionally, when the pressure was released to the ambient condition (R1 atm), the PL center was ≈140 nm redshifted and the PL intensity was slightly increased compared with the initial one, indicating some irreversible changes within the crystalline structures (Figure S8, Supporting Information).

To get a deepen insight into the pressure‐induced PL variations, more PL experiments were conducted at high pressure. The PL intensity of peak I and peak II increased continuously and exhibited a linear dependence on the excitation power density (Figure S9, Supporting Information). It suggested that the PL emission of high‐pressure [Pb_3_Br_4_][O_2_C(CH_2_)_2_CO_2_] is not related to surface defects.^[^ ^11^ ^]^ Meanwhile, the PL lifetimes of peak I and peak II are measured as 65.0 and 51.4 ns, respectively, which belong to the same order of magnitude (Figure S10, Supporting Information). Hence, the peak II should also derive from STE emission. Furthermore, in order to determine the emission origins of peak I and peak II, another 3D metal halide of [Pb_2_Br_2_][O_2_C(CH_2_)_4_CO_2_] was chosen for comparison.^[^ ^6^ ^]^ The [Pb_2_Br_2_][O_2_C(CH_2_)_4_CO_2_] is constructed by the PbBr_3_ units and adipates, which are also covalently bridged to form the ring channel structure (Figure S11, Supporting Information). The inter‐ and intra‐ octahedra distortions of Pb_2_Br_3_O_3_ lead to the one STE emission peak, which is similar to the emission peak I of [Pb_3_Br_4_][O_2_C(CH_2_)_2_CO_2_] at 1 atm (Figure S12, Supporting Information). Meanwhile, the structure distortion of Pb_2_Br_4_O_2_ octahedra in [Pb_3_Br_4_][O_2_C(CH_2_)_2_CO_2_] is closed to Pb_2_Br_3_O_3_ octahedra in [Pb_2_Br_2_][O_2_C(CH_2_)_4_CO_2_]. It suggests that the peak I of [Pb_3_Br_4_][O_2_C(CH_2_)_2_CO_2_] is related to the Pb_2_Br_4_O_2_ octahedra and the pressure‐induced peak II should be identified as the STE emission of Pb_2_Br_2_O_2_ tetrahedra.^[^ ^12^ ^]^ The pressure‐induced new STE emission from tetrahedral units is realized in 3D metal halides for the first time.

**Figure 2:**
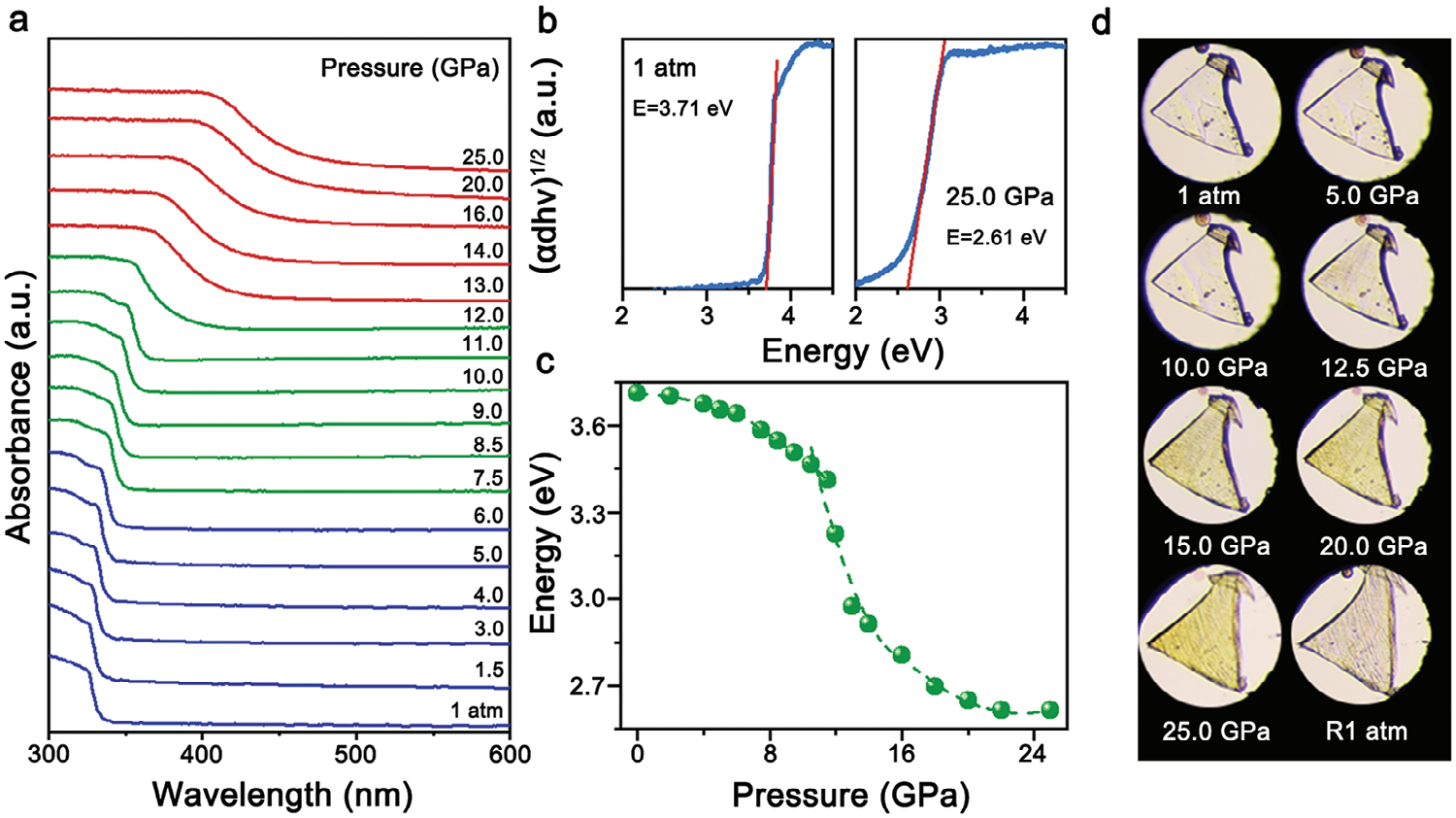
a) High‐pressure UV–vis absorption spectra of [Pb_3_Br_4_][O_2_C(CH_2_)_2_CO_2_]. b) Tauc plots at 1 atm (left) and 25.0 GPa (right). c) Bandgap changes with increasing pressure. d) Optical micrographs of [Pb_3_Br_4_][O_2_C(CH_2_)_2_CO_2_] crystal at selected pressures.

In situ high‐pressure UV–vis absorption spectroscopy, as a reliable method to trace the band structure variation, could provide further clues to understand the evolution of PL emission. At 1 atm, the [Pb_3_Br_4_][O_2_C(CH_2_)_2_CO_2_] adopted direct‐type bandgap, as evidenced by the first‐principles density‐functional calculation (Figure S13, Supporting Information). The valence band maximum primarily comprised of the 6s orbital of Pb, the 4p orbital of Br and the 2p

orbital of O. While the conduction band minimum was mainly dominated by the hybridization between the 6p orbital of Pb. With the function of high pressure, the absorption edge of [Pb_3_Br_4_][O_2_C(CH_2_)_2_CO_2_] was continuously redshifted (**Figure** **2**a). Up to 25.0 GPa, the bandgap value gradually decreased from 3.71 to 2.63 eV (Figure 2b,c). The crystal color gradually deepened as the narrow of bandgap is shown in optical micrographs (Figure 2d). Consistent with the PL variation, the pressure‐dependent bandgap value also revealed discontinued evolution at 7.5 GPa. Normally, the pressure‐triggered contractions of Pb─Br and Pb─O bonds are capable of enhancing the coupling among Pb‐6s, Br‐4p, and O‐2p orbitals, so as to reduce the bandgap.^[^ ^13^ ^]^ The contracted bonds with narrowed bandgap could contribute to the redshifts of PL emission.^[^ ^14^ ^]^ Meanwhile, compared with the absorption signal at 1 atm, the absorption edge at R1atm exhibited a ≈30 nm redshift, and the Stokes shift also increased to 281 nm, which should be ascribed to some irreversible variations in electronic structures (Figure S14, Supporting Information).

**Figure 3:**
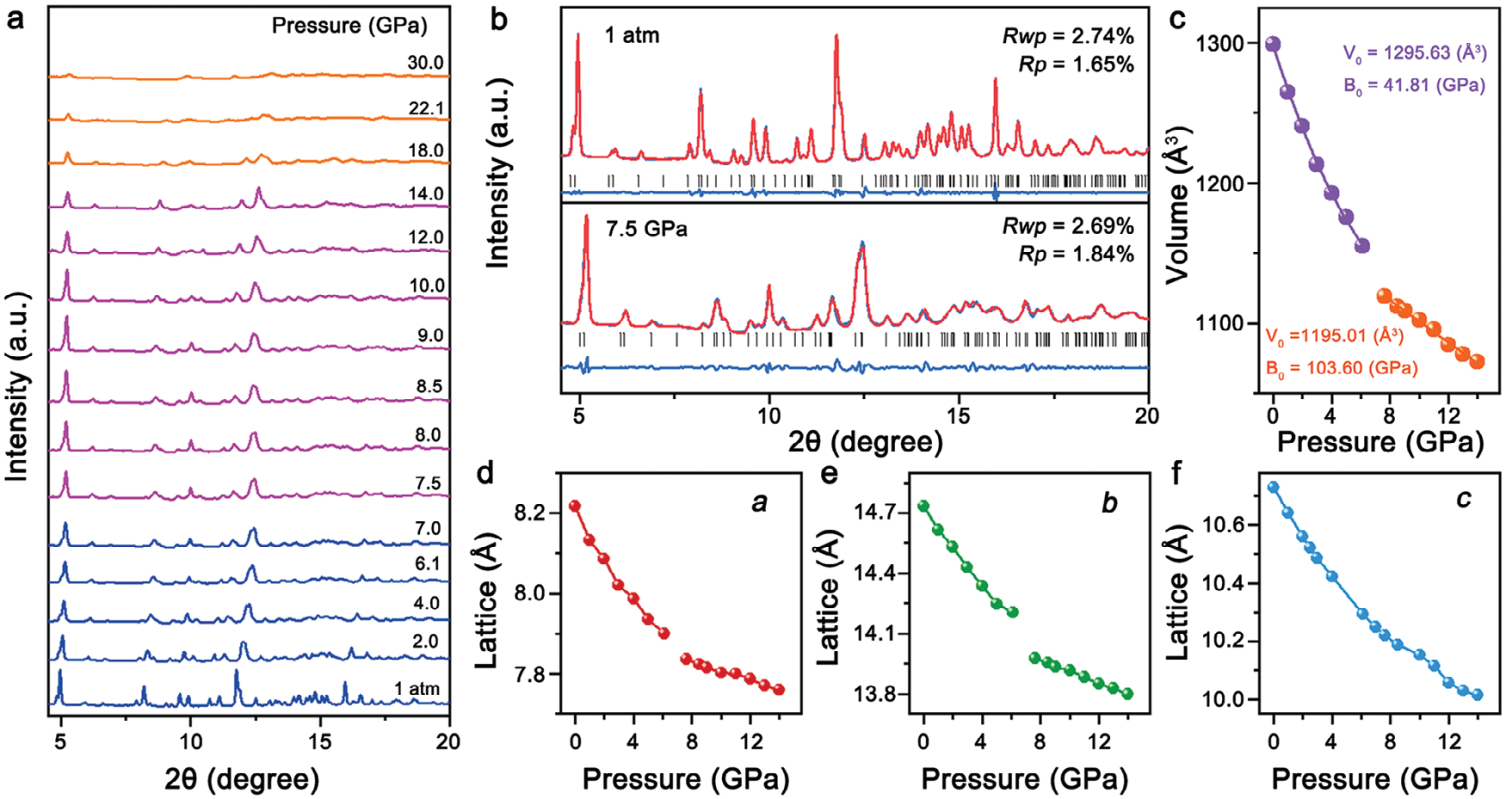
a) High‐pressure XRD patterns of [Pb_3_Br_4_][O_2_C(CH_2_)_2_CO_2_] with increasing pressure to 30 GPa. b) Rietveld refinements at (up)1 atm and (down) 7.5 GPa. c) High‐pressure evolution of lattice cell volume. Pressure‐dependent evolution of lattice constants of d) *a*, e) *b*, and f) *c* axes.

To deeply understand the correlation between structure and optical behavior, in situ synchrotron XRD experiments were carried out on [Pb_3_Br_4_][O_2_C(CH_2_)_2_CO_2_]. During compression, all the Bragg diffraction peaks were shifted toward higher angles without the emergence of new peaks (**Figure** **3**a). Below 6.1 GPa, the movement rate of diffraction peaks was relatively fast, indicating the easily compressed crystalline structure. While, the diffraction peaks moved slowly between 7.5 and 14.0 GPa, illustrating the enhanced lattice rigidity in this pressure range. Meanwhile, further compression above 14.0 GPa induced the obvious broadening and weakening of diffraction peaks, suggesting the decreased crystallinity of

[Pb_3_Br_4_][O_2_C(CH_2_)_2_CO_2_].^[^ ^15^ ^]^ At 30.0 GPa, the sample became almost amorphous, as evidenced by the disappeared diffraction signal. Furthermore, the overall intensity of diffraction signal was considerably weakened with the disappearance of some diffraction peaks at R1 atm, illustrating the partially reversible nature of structural amorphization (Figure S15, Supporting Information).

Based on the high‐pressure XRD patterns, Rietveld refinement was performed to reveal the specific changes in [Pb_3_Br_4_][O_2_C(CH_2_)_2_CO_2_] architecture (Figure 3a). At 1 atm, [Pb_3_Br_4_][O_2_C(CH_2_)_2_CO_2_] possessed orthorhombic structure with the lattice parameters of *a* = 8.22(3) Å, *b* = 14.73(2) Å, *c* = 10.73(2) Å, *β* = 90.00°, V = 1299.00(1) Å^3^ (Figure 3b). During the compression to 14.0 GPa, the *a* and *b* axes exhibited discontinuously decreasing at 7.5 GPa, while the contraction of *c*‐axis was successive (Figure 3d–f). In the pressure range of 1 atm–7.0 GPa, the compression rate of *a* axis was 0.0063 /GPa, which is higher than the 0.0060 /GPa of *b* axis (Figure S16, Supporting Information). Between 7.5 and 14.0 GPa, the *b*‐axis became easier to be compressed than *a*‐axis. These results disclosed that there are two contraction processes in high‐pressure [Pb_3_Br_4_][O_2_C(CH_2_)_2_CO_2_] structure. Notably, the bulk modulus (B_0_) increased from 41.81 to 103.60 GPa at the critical pressure of 7.5 GPa, which was consistent with the characteristics of isostructural phase transition (Figure 3c). Meanwhile, in situ high‐pressure Raman spectra and infrared absorption spectrometry experiments also declared this isostructural phase transition (Figures S17 and S18, Supporting Information).

**Figure 4:**
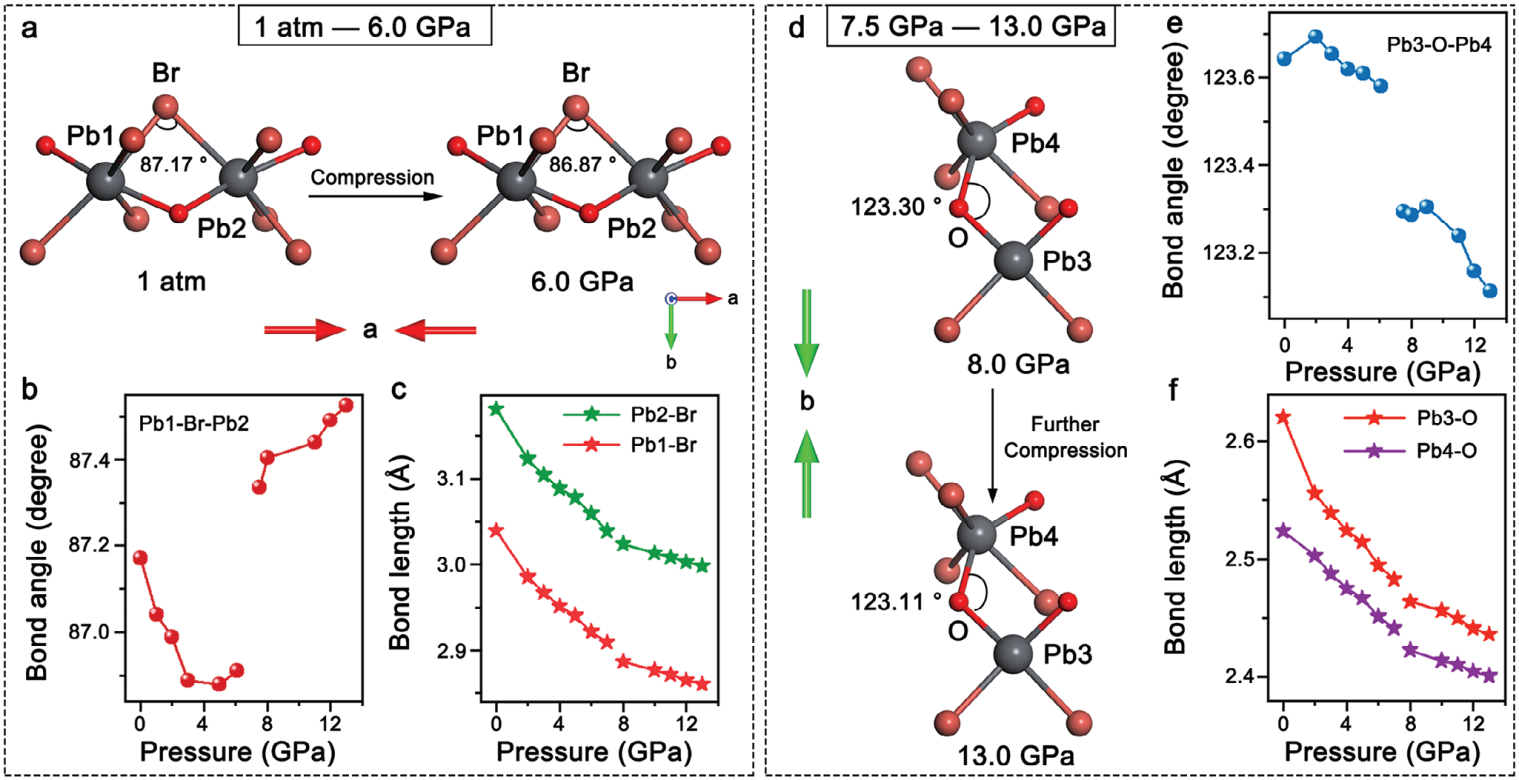
a) Schematic illustrations of Pb1‐Br‐Pb2 band angle within unit 1 at 1 atm and 6.1 GPa (up). The evolution of b) Pb1‐Br‐Pb2 band angle and c) Pb‐Br band length upon compression. d) The variations of Pb3‐O‐Pb4 band angle at 8.0 and 13.0 GPa within unit 2. The evolution of e) Pb3─O─Pb4 bond angle and f) the Pb─O bond length from 1 atm to 13.0 GPa.

Interatomic bond lengths and angles are important structural parameters that affect the orbital coupling and further determine the optical properties of [Pb_3_Br_4_][O_2_C(CH_2_)_2_CO_2_]. In the initial compression stage (<7.0 GPa), the lattice structure along *a* axis was significantly compressed, showing obviously decreased Pb1─Br─Pb2 bond angle in unit 1 and the hardly changed Pb3─O─Pb4 bond angle in unit 2 (**Figure** **4**a–c,e). The decreased Pb1─Br─Pb2 bond angle suggested the enhanced structural distortion between Pb_2_Br_4_O_2_ octahedra, which could increase the orbital overlaps and contribute to the redshifts and the slight enhancement of the STE emission of peak I. At 7.5 GPa, [Pb_3_Br_4_][O_2_C(CH_2_)_2_CO_2_] underwent isostructural phase transition and the unit 2 shrinkage along the *b* axis became more dominated (Figure 4d–f). The Pb1─Br─Pb2 bond angle gradually broadened with increasing pressure and reached the maximum at 13.0 GPa (Figure 4b). While the Pb3─O─Pb4 bond angle was significantly narrowed as the promoted inter‐polyhedral distortion, which should be conducive to the appearance of peak II. To quantitatively identify the distortion degree of Pb_2_Br_2_O_2_ tetrahedra and Pb_2_Br_4_O_2_ octahedra, the polyhedral variance of $\sigma_{oct}^{2}$ was calculated with increasing pressure. During compression, the $\sigma_{oct}^{2}$ of Pb_2_Br_4_O_2_ octahedra was barely changed below 3.0 GPa and then gradually increased up to 6.0 GPa. The increased $\sigma_{oct}^{2}$ implies the

promoted Pb_2_Br_4_O_2_ octahedral distortion, which should be responsible for the enhanced intensity of peak I (Figures S19 and S20, Supporting Information).^[^ ^16^ ^]^ Note that the $\sigma_{oct}^{2}$ of Pb_2_Br_2_O_2_ tetrahedra remained almost unchanged in this pressure interval. At 8.0 GPa, the $\sigma_{oct}^{2}$ of both Pb_2_Br_4_O_2_ octahedra and Pb_2_Br_2_O_2_ tetrahedra were dramatically increased, suggesting the enhanced lattice distortion around the isostructural phase transition. The sudden distortion of Pb_2_Br_2_O_2_ tetrahedra would enhance the electron‐phonon coupling strength to contribute to the STE emission of peak II.^[^ ^5a^ ^]^ Above 8.0 GPa, the continuously increased $\sigma_{oct}^{2}$ implied the further polyhedral distortion, promoting the STE emission. In addition, the contraction of the Br─Pb─O bond angle in Pb_2_Br_4_O_2_ octahedra induced the bandgap narrowing continuously upon compression and the change of the decay rate (Figures S21–S25, Supporting Information). It verifies that the intra‐ and inter‐ polyhedral distortion is an important factor to affect the optical properties of [Pb_3_Br_4_][O_2_C(CH_2_)_2_CO_2_].

**Figure 5:**
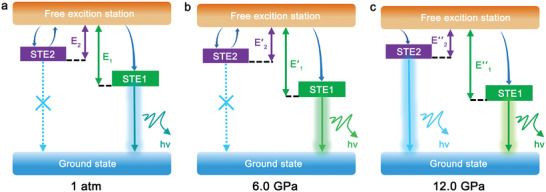
Illustration of the energy level diagram and emission mechanism at a) 1 atm, b) 6.0 GPa, and c) 12.0 GPa. STE1: self‐trapped emission state 1, STE2: self‐trapped emission state 2, E_1_, E_2_: the self‐trapping depths of STE1 and STE2.

Based on the high‐pressure PL and synchrotron XRD experiments, the mechanism of optical variations of [Pb_3_Br_4_][O_2_C(CH_2_)_2_CO_2_] should be explained as follows (**Figure** **5**). Within [Pb_3_Br_4_][O_2_C(CH_2_)_2_CO_2_], the photoactive components of Pb_2_Br_4_O_2_ octahedra and Pb_2_Br_2_O_2_ tetrahedra provide the distinct sets of self‐trapped states, which are identified as STE1 and STE2. Upon excitation, the electrons are first excited from ground state to FE state, and then transferred to the STE states with instantaneous excited‐state distortion and relaxation. At 1 atm, the Pb_2_Br_2_O_2_ tetrahedral distortion is insufficient to provide enough electron‐phonon coupling for trapping the FEs to STE2 (Figure 5a). The excitons could be easily detrapped from the STE2 state to the FE state by thermal activation. Hence, the ambient [Pb_3_Br_4_][O_2_C(CH_2_)_2_CO_2_] only exhibits one STE emission peak from STE1 (Pb_2_Br_4_O_2_ octahedra). With increasing pressure to 6.0 GPa, the enhanced Pb_2_Br_4_O_2_ octahedral distortion with decreased Pb1‐Br‐Pb2 band angle promotes the overlap of adjacent electron clouds and facilitates the electron‐phonon coupling effects. The accordingly elevated activation energy for excitonic detrapping endows more excitons to be trapped within STE1 and thus improves the intensity of peak I. As for the STE2, the distortion of Pb_2_Br_2_O_2_ tetrahedra only fluctuates slightly in this pressure interval that the STEs are still unable to be bound for radiative transition (Figure 5b). The movement of STE emission under pressure is mainly attributed to the transformation of lattice relaxation energy ***E***_***LR***_, which related to FWHM (∆) by the relationship $\Delta \propto \sqrt{2E_{LR}K_{B}T}$.^[^ ^17^ ^]^ In this interval, the gradual widening of FWHM along with compression induces the increase of ***E***_***LR***_. This triggers a gradual deepening of the trap depth E_1_ and thus STE1 emission redshift. Upon further compression, the isostructural phase transition at 7.5 GPa results in the enhanced distortion of Pb_2_Br_2_O_2_ tetrahedra (increased $\sigma_{oct}^{2}$, as well as the distortion between Pb_2_Br_4_O_2_ octahedra and Pb_2_Br_2_O_2_ tetrahedra (decreased Pb─O─Pb bond angle). The increases of electron cloud overlaps and detrapping activation energy of STEs enable more excitons to relax and bond toward to STE2. Then, the new STE emission from Pb_2_Br_2_O_2_ tetrahedra is appeared at this critical pressure. In addition, the continued lattice distortion above 7.5 GPa leads to the further enhancement of electron‐phonon coupling strength, and the decreasing of ***E***_***LR***_ results in the shallow of the self‐trapped depth E_1_ and E_2_, thus resulting in the emission enhancement and movement with obvious color and brightness changes (Figure 5c).

## 3 Conclusion

To sum up, the optical and structure properties of 3D metal halide [Pb_3_Br_4_][O_2_C(CH_2_)_2_CO_2_] were investigated at high pressure. Dual STE emission and obvious emission enhancement were implemented upon compression. At 7.5 GPa, an isostructural phase transition occurred in [Pb_3_Br_4_][O_2_C(CH_2_)_2_CO_2_], accompanied by a new fluorescence peak. The pressure‐induced new emission peak is ascribed to the radiative recombination of STEs formed by the considerable distortion of Pb_2_Br_2_O_2_ tetrahedra and the increased distortion between Pb_2_Br_4_O_2_ octahedra and Pb_2_Br_2_O_2_ tetrahedra. Our studies not only elucidate the underlying emission mechanism of the STE emission for ambient [Pb_3_Br_4_][O_2_C(CH_2_)_2_CO_2_], but also construct the relationship between structure and optical behavior. It is expected this work could provide new strategies for improving emission intensity and effectively modulating the emission color of 3D metal halides.

## Conflict of Interest

The authors declare no conflict of interest.

## Supporting information

Supporting Information

## Data Availability

The data that support the findings of this study are available in the supplementary material of this article.
