# Supplementary material for: Intense Broadband Emission in the Unconventional 3D Hybrid Metal Halide via High‐Pressure Engineering
Source: Adv Sci (Weinh). 2023 Dec 24;11(10):2306937. doi: 10.1002/advs.202306937 (PMC10933603; doi:10.1002/advs.202306937)
Supplement: Supplementary file 1 — Supporting Information [file ADVS-11-2306937-s001.pdf]

ADVANCED SCIENCE
Open Access

Supporting Information

for *Adv. Sci.*, DOI 10.1002/advs.202306937

Intense Broadband Emission in the Unconventional 3D Hybrid Metal Halide via High-Pressure Engineering

Xuening Sun, Min Wu, Xihan Yu, Qian Li, Guanjun Xiao, Kai Wang and Bo Zou*

Supporting Information
©Wiley-VCH 2021
69451 Weinheim, Germany

Intense Broadband Emission in the Unconventional 3D Hybrid Metal Halide via High Pressure Engineering

Xuening Sun, Min Wu, Xihan Yu, Qian Li, Guanjun Xiao*, Kai Wang and Bo Zou

Abstract: Developing hybrid metal halides with self-trapped exciton (STE) emission is a powerful and promising approach to achieve single-component phosphors for wide-color-gamut display and illumination. Nevertheless, it is difficult to generate STEs and broadband emission in the classical and widely used three-dimensional (3D) systems, owing to the great structural connectivity of metal-halogen networks. Here, high pressure was implemented to achieve dual emission and dramatical emission enhancement in 3D metal halide of $[Pb_3Br_4][O_2C(CH_2)_2CO_2]$. The pressure-induced new emission is ascribed to the radiation recombination of STEs from the $Pb_2Br_2O_2$ tetrahedra with the promoted distortion through the isostructural phase transition. Furthermore, the wide range of emission chromaticity can be regulated by controlling the distortion order of different polyhedral units upon compression. This work not only construct the relationship between structure and optical behavior of $[Pb_3Br_4][O_2C(CH_2)_2CO_2]$, but also provide new strategies for optimizing broadband emission toward potential applications in solid-state lighting.

DOI: 10.1002/anie.2021XXXXX

Experimental Procedures

Material synthesis

The $[Pb_3Br_4][O_2C(CH_2)_2CO_2]$ crystals were synthesized according to the method that has been previously reported in the literature. Lead bromide ($PbBr_2$, 2.0 mmol, 0.7340g, Adamas, 99.9%), succinic acid disodium salt ($NaO_2C(CH_2)_2CO_2Na$, 4.0 mmol, 0.6482g, Sigma-Aldrich, 99.0%), perchloric acid ($HClO_4$, 5.56 mmol, 460 μL, Greagent, 70%), and deionized water (16 mL) were loaded into a 25 mL Teflon-lined autoclave reactor. The solution was stirred for 15 min for sufficient dispersion. The autoclave was then sealed into a stainless steel vessel and heated at 175 °C for 48 h. After incubation, slow-cooling of the autoclaves at the rate of 10° C/h to room temperature yielded colorless block-shaped crystals. The crystals were washed with ethanol and deionized water, and dried overnight under 30 Pa pressure with vacuum.

In situ high-pressure experiments

High-pressure experiments were conducted using a diamond anvil cell (DAC) with 400 μm diameter culets. A T301 stainless steel gasket with a 150 μm hole and 45 μm thickness was served as the sample chamber. The sample was loaded into the sample chamber along with a ruby ball to determine pressure according to the ruby fluorescence technique. *In situ* high-pressure optical absorption, photoluminescence (PL) and X-ray diffraction (XRD) experiments, silicone oil was applied as pressure-transmitting medium.

The *in situ* high-pressure PL measurements were measured using the 355 nm line of a UV DPSS laser. *In situ* high-pressure UV-Vis absorption measurements were performed by a deuterium-halogen light source. The PL and absorption microphotographs were captured with a camera (Canon Eos 5D mark II) equipped on a microscope (Ecilipse TI-U, Nikon). The optical fiber spectrometer is an Ocean Optics QE65000 spectrometer.

The *in situ* high-pressure time-resolved PL measurements were performed using a 375 nm pulsed diode laser (LDH-P-C-375B, 40 ps) as excitation source. A 20× ultraviolet objective lens was utilized to project the incident laser onto the sample and collet the

backscattered emission signal. The PL signal was directed into the 500 mm focal length grating spectrograph (HRS-500 MS), where a PMT together with a time correlated single photon counting electronics (TimeHarp 260 PICO) was used to detect the PL kinetics.
The time resolved PL decay curves were fitted by the double exponential function:

$$I(t) = I_0 + A_1 \times \exp\left(-\frac{t}{\tau_1}\right) + A_2 \times \exp\left(-\frac{t}{\tau_2}\right) \quad (1)$$

The average lifetime τ was calculated by the follow equation:

$$\tau = \frac{(A_1 \times \tau_1{}^2) + (A_2 \times \tau_2{}^2)}{(A_1 \times \tau_1) + (A_2 \times \tau_2)} \quad (2)$$

The *in situ* high-pressure angle-dispersive XRD measurements (λ = 0.6199 Å) were performed at BL15U1 at the Shanghai Synchrotron Radiation Facility (SSRF). Before the experiments, CeO_2 was used for geometry calibration. All the high-pressure experiments were conducted at room temperature. The diffraction patterns were integrated into one-dimensional profile using Fit2D program. The Reflex module combined in Materials Studio was applied for Rietveld refinement. The pressure-volume data were fitted by the third-order Birch-Murnaghan equation of state as follows:

$$\mathrm{P} = \frac{3}{2} B_0 \times \left[\left(\frac{V}{V_0}\right)^{-\frac{7}{3}} - \left(\frac{V}{V_0}\right)^{-\frac{5}{3}}\right] \times \left\{1 + \frac{3}{4}(B'_0 - 4) \times \left[\left(\frac{V}{V_0}\right)^{-\frac{2}{3}} - 1\right]\right\} \quad (3)$$

where V_0 is the zero-pressure volume, B_0 is the bulk modulus at ambient pressure, and B'_0 is a parameter for the pressure derivative.

The *in situ* high-pressure Raman measurements were carried out by a Raman spectrometer (iHR 550, Syncerity, Horiba Jobin Yvon) with a 785 nm laser excitation. Liquid nitrogen was selected as the pressure transfer medium for the experiments. The *in situ* high-pressure IR absorption measurements were conducted using a Bruker Vertex 70 V FT-IR spectrometer (BRUKER OPTIK GMBH, Germany). Potassium bromide is used as a pressure transfer medium for testing.

To evaluated the distortion degree of the $Pb_2Br_4O_2$ octahedra and $Pb_2Br_2O_2$ tetrahedra based on the polyhedral variance $\sigma_{oct}{}^2$ by the Pb-X (X = Br and O) bond lengths.

$$\sigma_{oct}{}^2 = \frac{1}{n}\sum_{i=1}^{n}(\alpha_i - \alpha)^2 \quad (4)$$

Where n is the number of Pb-X lengths in an octahedron or tetrahedron, α_i is the Pb-X length and α is the average bond length of octahedra and tetrahedra.

First-Principle Calculations.

Electronic band structures were calculated using pseudopotential plane-wave methods based on density functional theory implemented in the CASTEP package. The starting structure was obtained from the Cambridge Structure Database. The plane-wave cutoff energy of 500 eV and Monkhorst-Pack grid for the electronic Brillouin zone integration was 3 × 3× 2. The self-consistent field (SCF) tolerance was set as 5.0 × 10^{-6} eV/atom. The convergence thresholds between optimization cycles for maximum force, maximum stress and maximum displacement are set as 0.01 eV/Å, 0.02 GPa, and 5.0 ×10^{-4} Å, respectively.

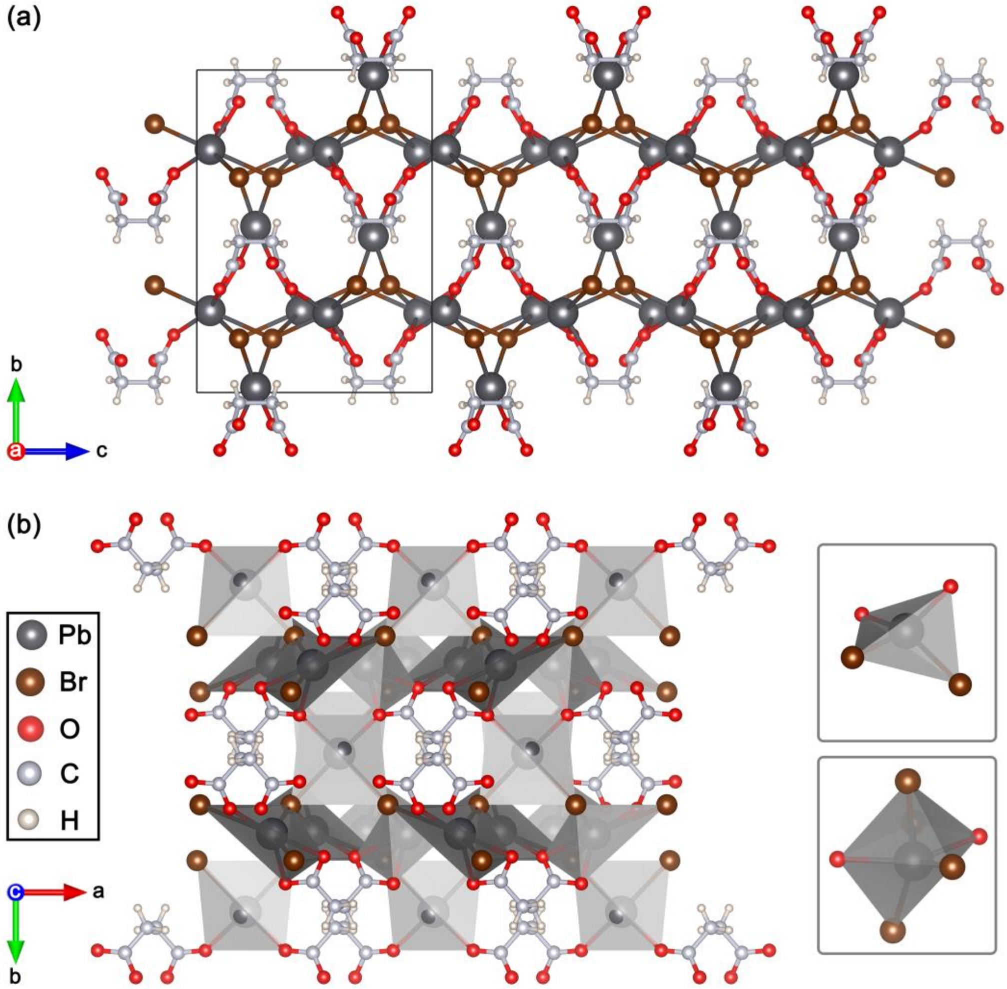

Figure S1. The crystalline structure of $[Pb_3Br_4][O_2C(CH_2)_2CO_2]$ viewed perpendicular to the (a) *a* axis and (b) *c* axis. The insets illustrate the different units of $Pb_2Br_4O_2$ octahedra and $Pb_2Br_2O_2$ tetrahedra.

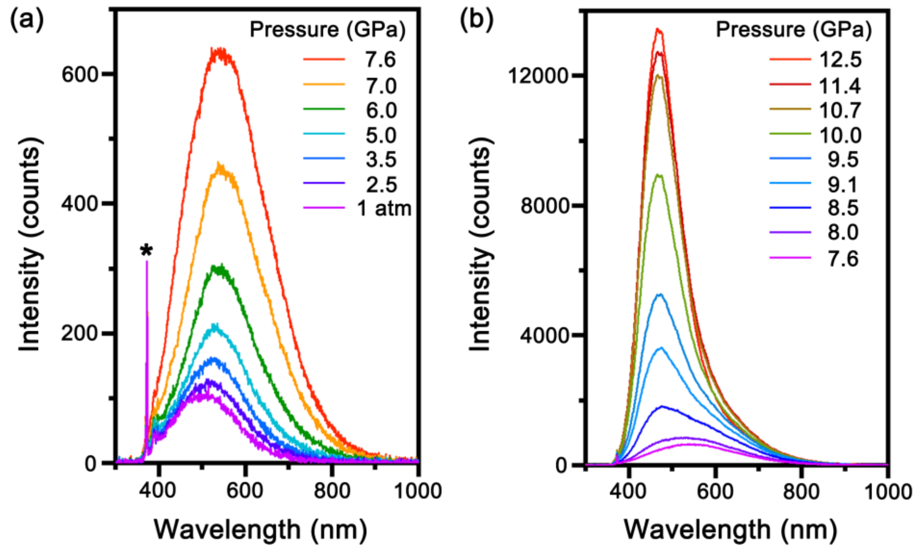

Figure S2. High-pressure PL spectra of $[Pb_3Br_4][O_2C(CH_2)_2CO_2]$ detected at selected pressures (a) from 1 atm to 7.6 GPa and (b) from 7.6 GPa to 12.5 GPa. (The signal marked with ***** comes from a diamond).

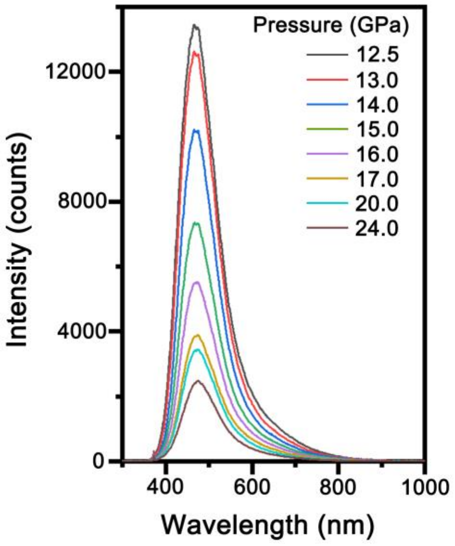

Figure S3. High-pressure PL spectra of $[Pb_3Br_4][O_2C(CH_2)_2CO_2]$ measured at selected pressures from 12.5 GPa to 24.0 GPa.

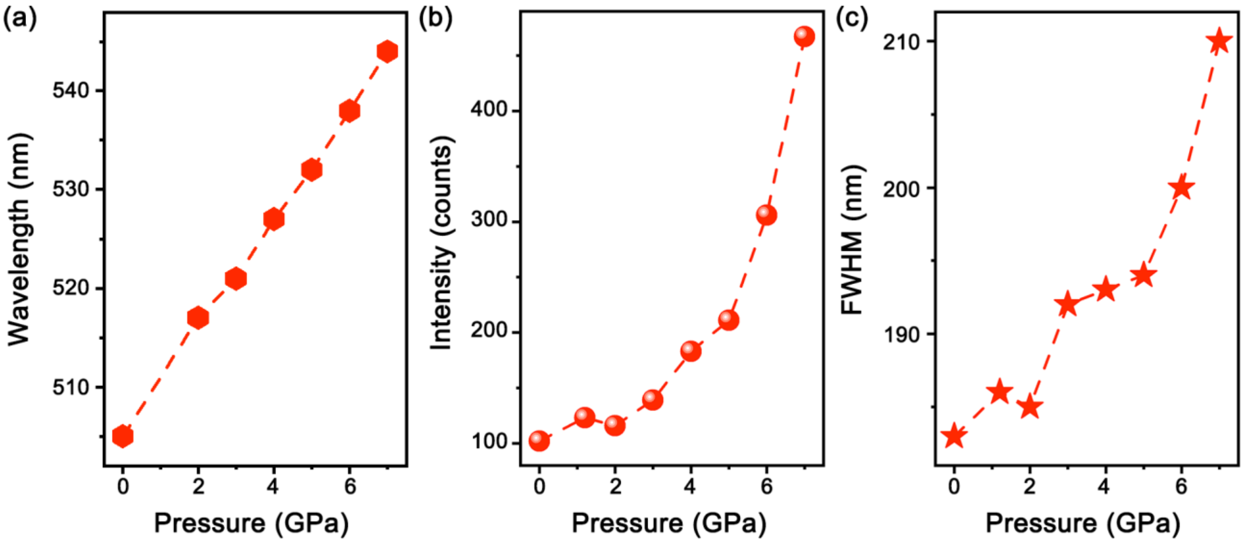

Figure S4. (a) PL location, (b) intensity and (c) full width at half-maximum (FWHM) of peak I with increasing pressure from 1 atm to 7.0 GPa.

Table S1. Type of emission and ratio of the maximum PL intensity via pressure (I_t) to the initial PL intensity (I_0) in 3D metal halides.

	Type of emission	I_t/I_0	Refs.
$CsPbBr_3$	band-edge emission	1	1
$CsPbBr_3:Mn^{2+}$	band-edge emission, Mn-related emission, STE emission	1.3	2
$CsPbCl_3$	band-edge emission	1	3
$CsPbCl_3:Eu^{3+}$	band-edge emission, Eu-related emission	3	3
$CsPbCl_3:Mn^{2+}$	band-edge emission, Mn-related emission	1.2	4
$FAPbBr_3$	band-edge emission	1	5
$CD_3ND_3PbI_3$	band-edge emission	3	6
$CH_3NH_3PbI_3$	band-edge emission	1.5	6
$Gd_2ZnTiO_6:Mn^{4+}$	Mn-related emission	1	7
$Cs_2NaBiCl_6$	STE emission	2.5	8
$Cs_2NaBiCl_6:Mn^{2+}$	Mn-related emission	3.5	8
$Cs_2AgBiCl_6$	STE emission	1.5	9
$Cs_2Na_{0.4}Ag_{0.6}InCl_6$	STE emission	1.5	10
**$[Pb_3Br_4][O_2C(CH_2)_2CO_2]$**	**STE emission**	**130**	**This work**

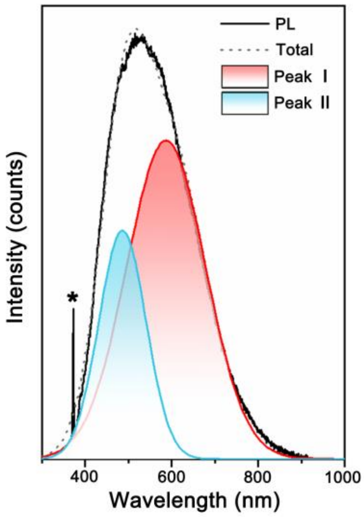

Figure S5. PL spectra of $[Pb_3Br_4][O_2C(CH_2)_2CO_2]$ at 8.0 GPa. (The signal marked with ***** comes from a diamond).

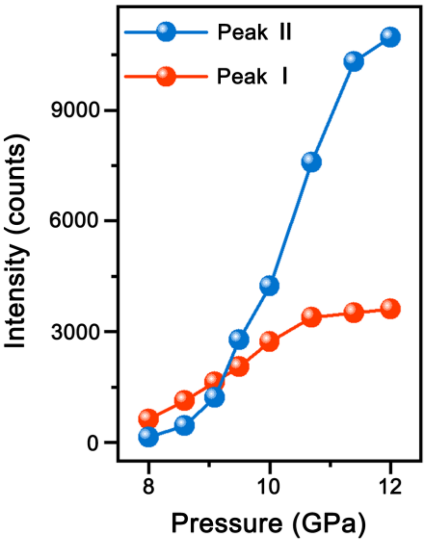

Figure S6. PL intensity of peak I and peak II upon compression from 8.0 GPa to 12.0 GPa.

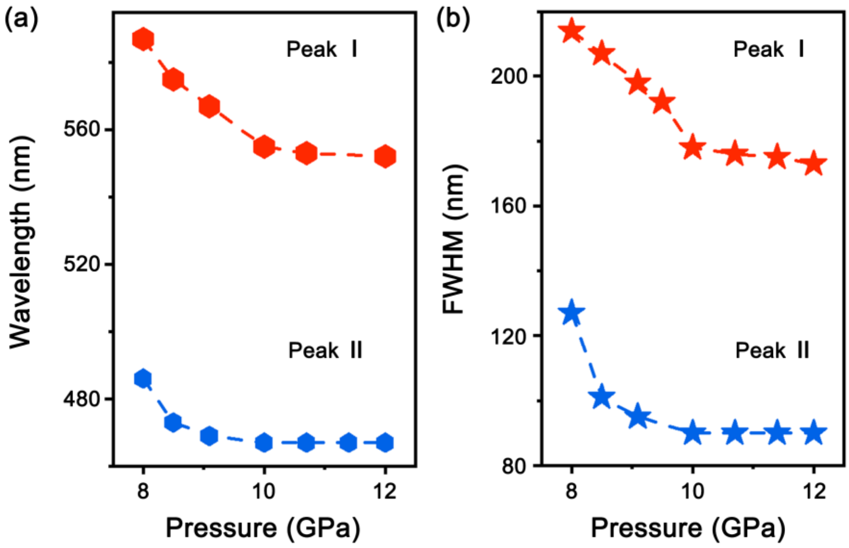

Figure S7. (a) PL Location and (b) Full width at half-maximum (FWHM) of peak I and peak II with increasing pressure from 8.0 GPa to 12.0 GPa.

Table S2. Commission Internationale de l´ Eclairage (CIE) coordinates of $[Pb_3Br_4][O_2C(CH_2)_2CO_2]$ at different pressure points.

Pressure (GPa)	CIE (x, y)	Pressure (GPa)	CIE (x, y)
1 atm	(0.27, 0.35)	7.6	(0.34, 0.39)
0.3	(0.28, 0.36)	8.0	(0.33, 0.38)
0.6	(0.29, 0.37)	8.5	(0.22, 0.34)
0.9	(0.27, 0.38)	9.1	(0.26, 0.30)
1.2	(0.29, 0.38)	9.5	(0.25, 0.29)
1.5	(0.29, 0.37)	10.0	(0.22, 0.26)
2.0	(0.29, 0.38)	10.7	(0.21, 0.26)
2.5	(0.29, 0.37)	11.4	(0.27, 0.35)
3.0	(0.30, 0.38)	12.0	(0.21, 0.25)
3.5	(0.31, 0.39)	13.0	(0.19, 0.24)
4.0	(0.31, 0.40)	14.0	(0.20, 0.24)
5.0	(0.32, 0.40)	15.0	(0.20, 0.25)
6.0	(0.34, 0.41)	18.5	(0.21, 0.27)
7.0	(0.35, 0.41)	24.0	(0.20, 0.27)

Table S3. Correlated color temperature (CCT) of $[Pb_3Br_4][O_2C(CH_2)_2CO_2]$ at different pressure points.

Pressure (GPa)	CCT (K)	Pressure (GPa)	CCT (K)
1 atm	8684	7.6	5155
0.3	8160	8.0	5651
0.6	7296	8.5	7549
0.9	8152	9.1	11425
1.2	7235	9.5	15273
1.5	7315	10.0	44493
2.0	7261	10.7	191497
2.5	7186	11.4	201167
3.0	6715	12.0	431391
3.5	6482	13.0	950000
4.0	6167	14.0	950000
5.0	5832	15.0	950000
6.0	5287	18.5	76627
7.0	4944	24.0	90257

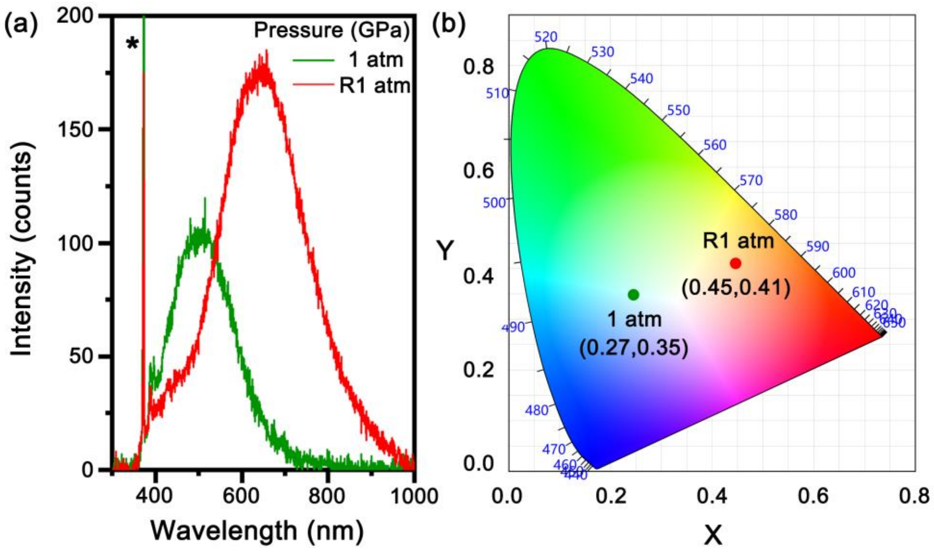

Figure S8. (a) PL spectra and (b) CIE coordinates between ambient condition and decompression. (The signal marked with ***** comes from a diamond).

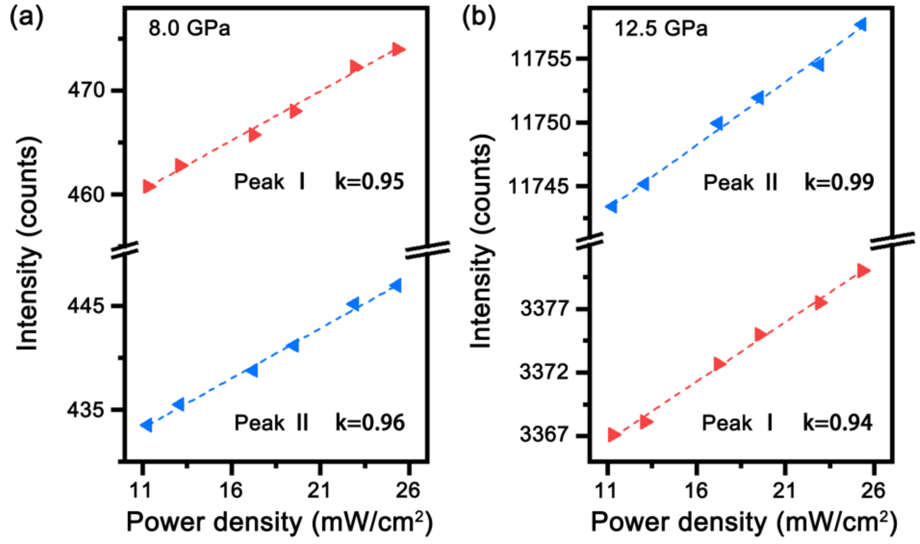

Figure S9. PL intensity of Peak I and Peak II at (a) 8.0 GPa, and (b) 12.5 GPa as a function of Power density, which is the "k" means the slope of PL intensity as a function of power density.

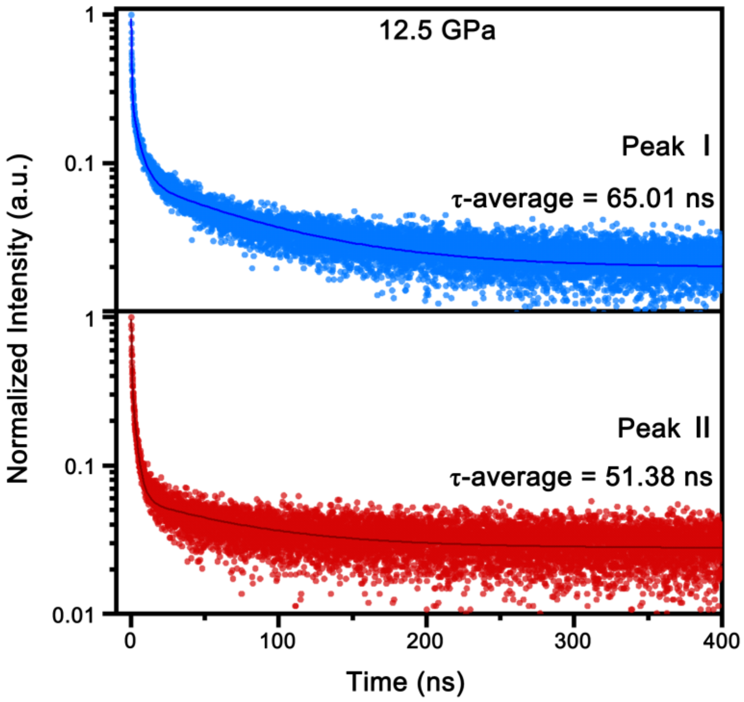

Figure S10. Normalized time-resolved PL decay curves of Peak I and Peak II at 12.5 GPa.

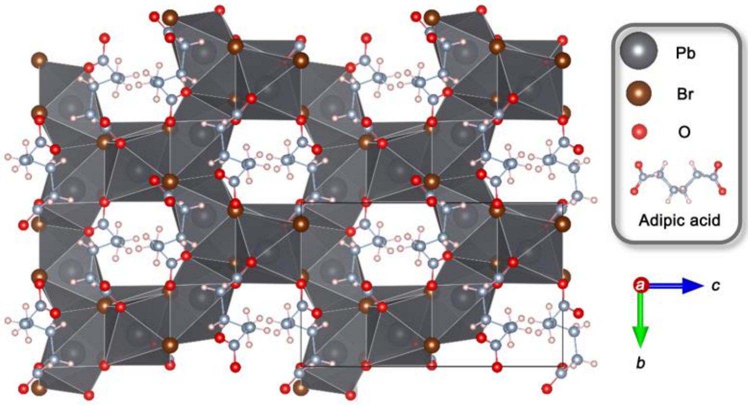

Figure S11. Crystalline structure of $[Pb_2Br_2][O_2C(CH_2)_4CO_2]$ viewed perpendicular to the *a* axis (up).

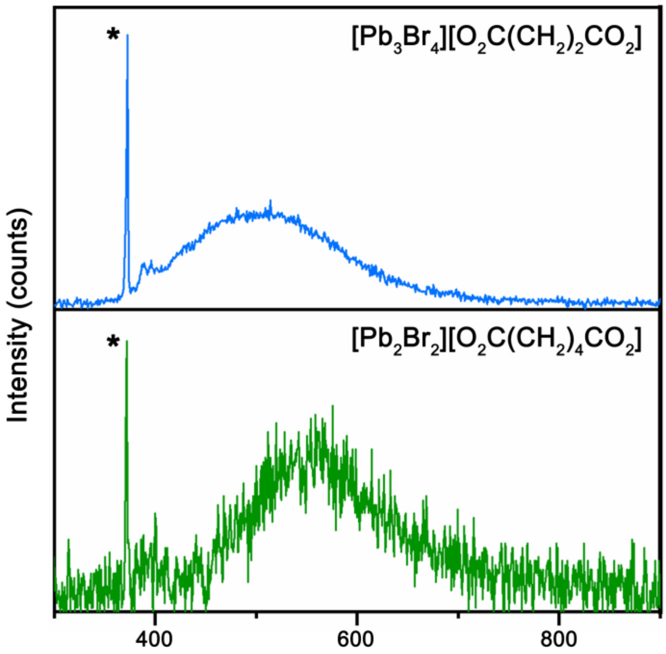

Figure S12. PL spectra of $[Pb_3Br_4][O_2C(CH_2)_2CO_2]$ and $[Pb_2Br_2][O_2C(CH_2)_4CO_2]$ at ambient conditions under UV irradiation of 355 nm. (The signal marked with ***** comes from a diamond).

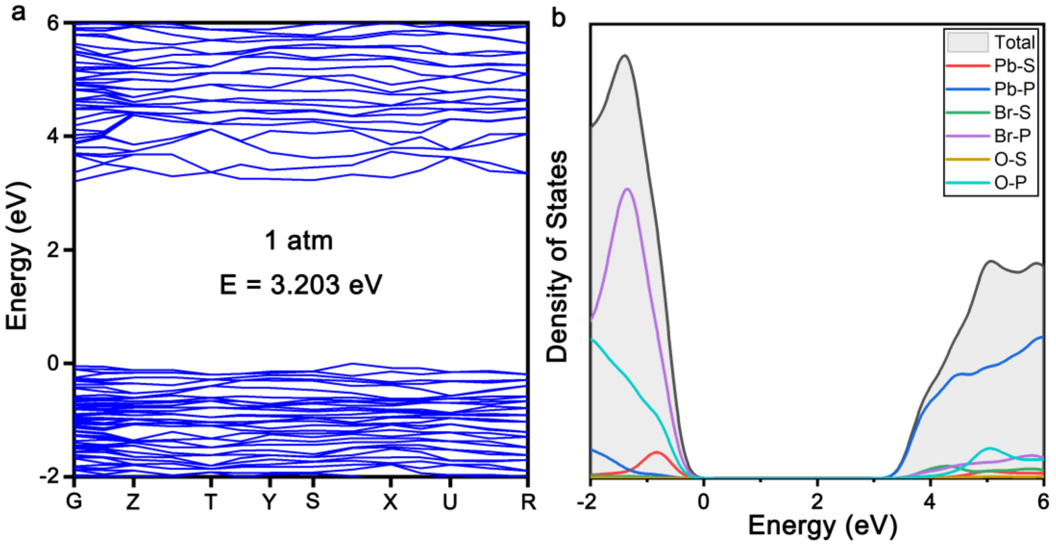

Figure S13. Calculated band structure and projected density of states of $[Pb_3Br_4][O_2C(CH_2)_2CO_2]$ at ambient conditions.

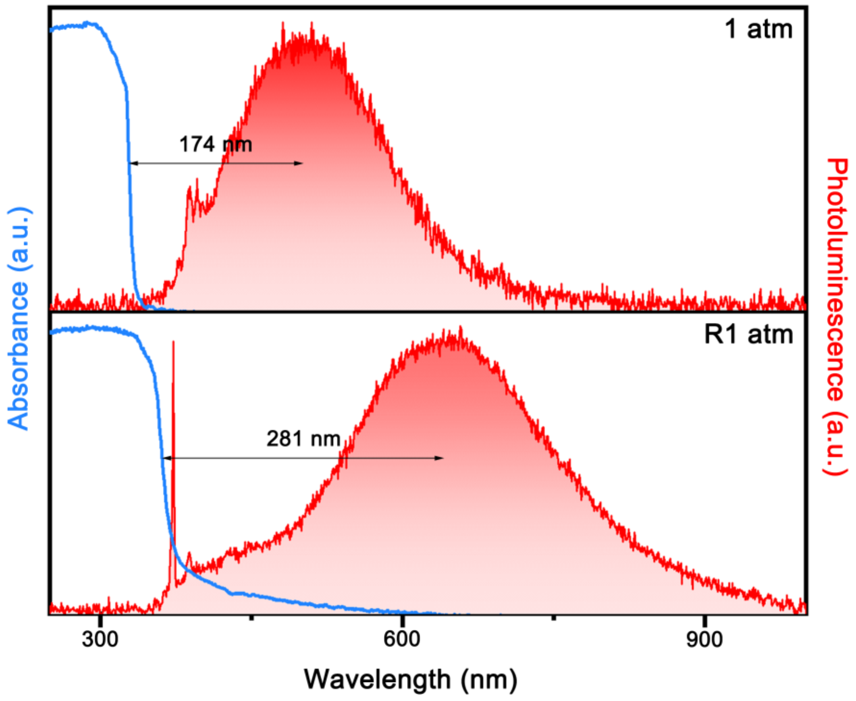

Figure S14. UV-Vis absorption (blue) and emission (red) spectra (a) 1 atm and (b) R1 atm.

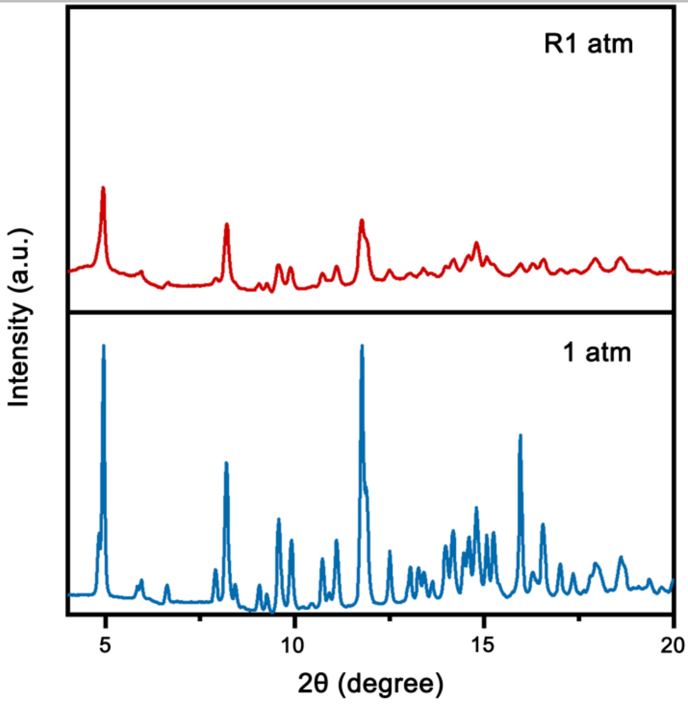

Figure S15. XRD patterns of $[Pb_3Br_4][O_2C(CH_2)_2CO_2]$ before and after pressure treatment.

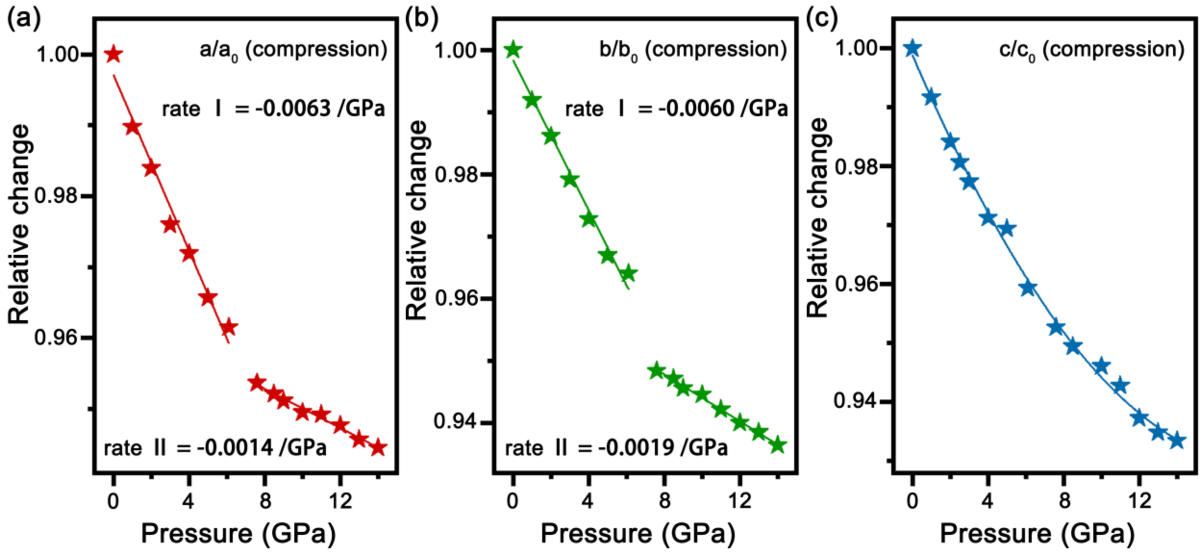

Figure S16. Pressure-dependent relative compression rates of (a) *a* axis, (b) *b* axis, (c) *c* axis.

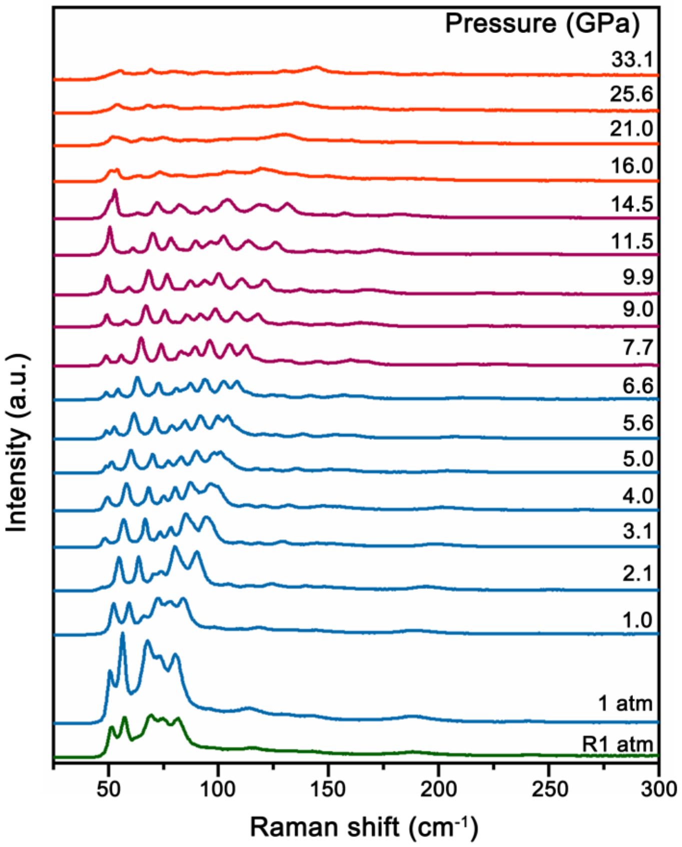

Figure S17. Raman spectra of $[Pb_3Br_4][O_2C(CH_2)_2CO_2]$ at selected pressure.

To track the evolution of the vibrational modes of $[Pb_3Br_4][O_2C(CH_2)_2CO_2]$, we carried out high-pressure Raman experiment. The low-frequency Raman vibrational modes (50-100 cm^{-1}) are assigned to the Pb-Br inorganic structure.[1] With the increase of pressure, Pb-Br vibrational modes showed evident blueshifts and the lattice modes below 50 cm^{-1} moved towards the detectable region, ascribed to the lattice contraction. Upon compression to 7.7 GPa, the shift rates of all vibration bands began to slow down without profile variation, indicating the increased rigidity of inorganic structure and the consequent isostructural phase transition. Under further compression to 16.0 GPa, most vibration peaks were widened and gradually disappeared, which should be ascribed to the seriously distorted inorganic structure.

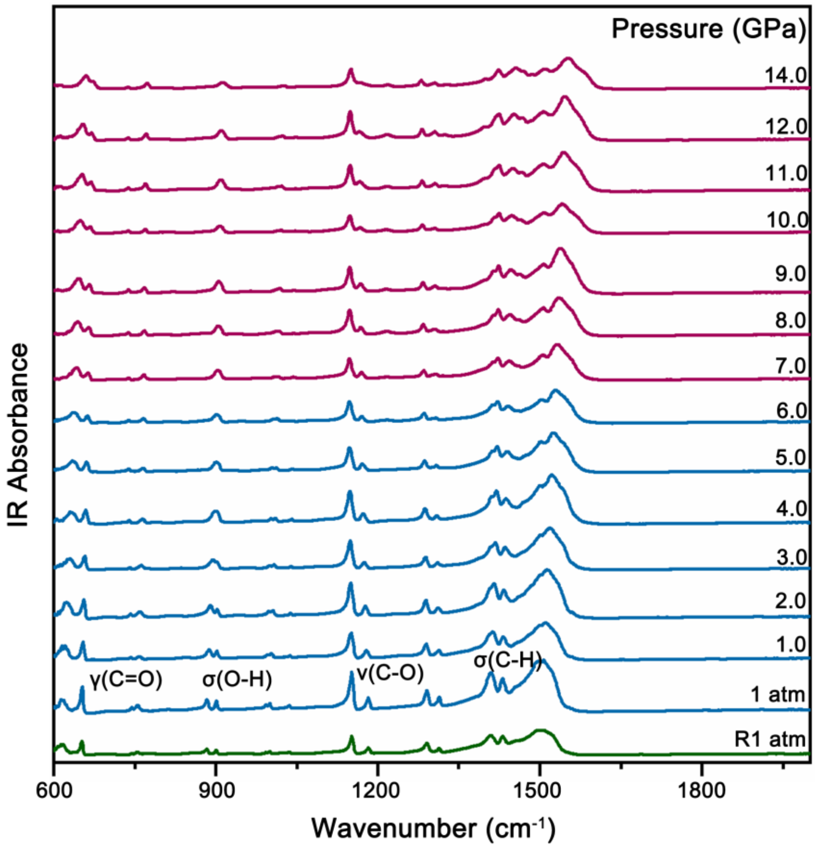

Figure S18. Infrared spectra of $[Pb_3Br_4][O_2C(CH_2)_2CO_2]$ at selected pressure.

High-pressure infrared absorption spectrometry was measured to investigate the behaviors of organic layer upon compression. The infrared absorption peaks were mainly concentrated in 600-1800cm^{-1}, including the out of plane C=O deformation vibration (617 and 650 cm^{-1}), the O-H bending mode (880 and 900 cm^{-1}), the C-O stretching mode (1150 and 1180 cm^{-1}), and the C-H bending mode (1400 cm^{-1}).[2] During the compression process, the peaks continued to move towards the high wavenumber region. After 7.0 GPa, the movement rates of infrared absorption peaks slowed down, accompanied by the peaks broadening and weakening. It is consistent with the change trend of inorganic structure, indicating that the behavioral changes of organic parts are closely related to the structural changes of inorganic structure.

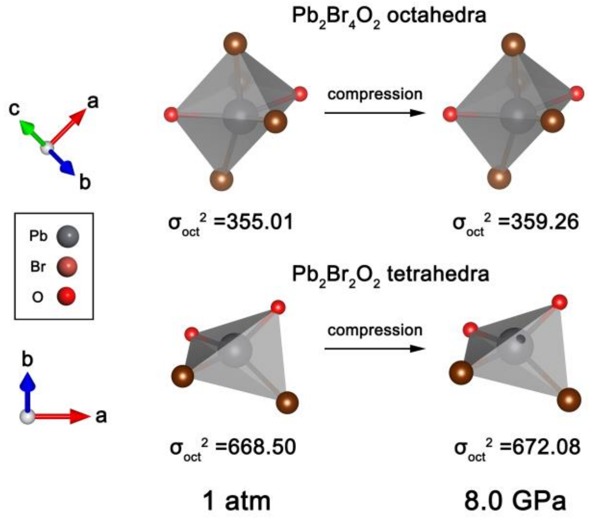

Figure S19. Distortion of $Pb_2Br_4O_2$ octahedra and $Pb_2Br_2O_2$ tetrahedra at 1 atm and 8.0 GPa.

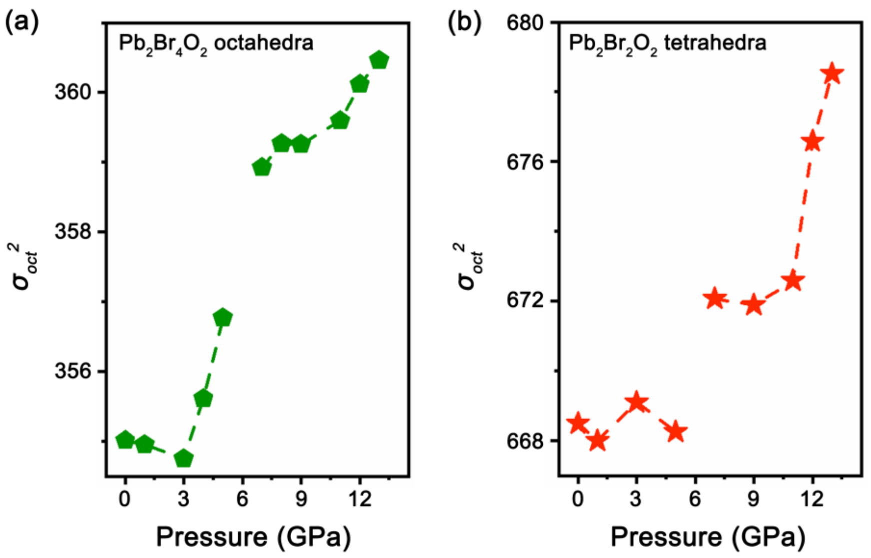

Figure S20. Distortion of $Pb_2Br_4O_2$ octahedra and $Pb_2Br_2O_2$ tetrahedra with increasing pressure.

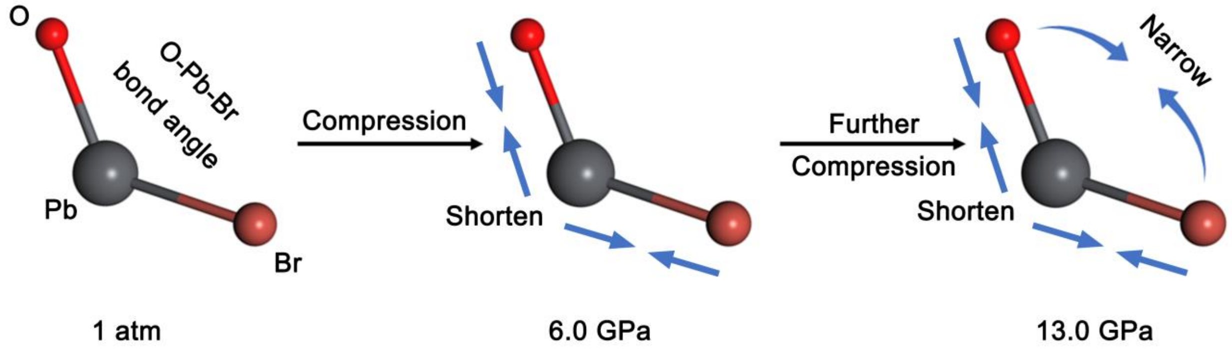

Figure S21. Schematic illustration of Br-Pb-O unit under pressure.

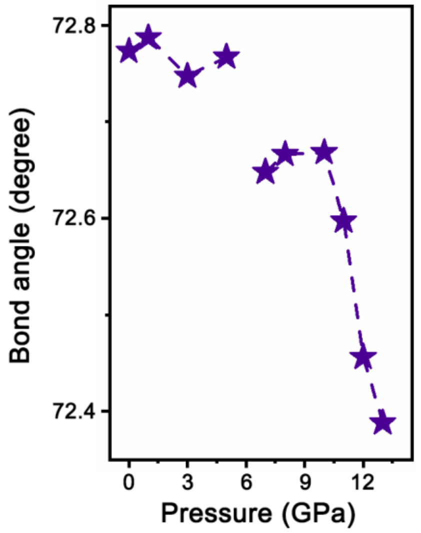

Figure S22. Evolution of bond angle of Br-Pb-O upon compression.

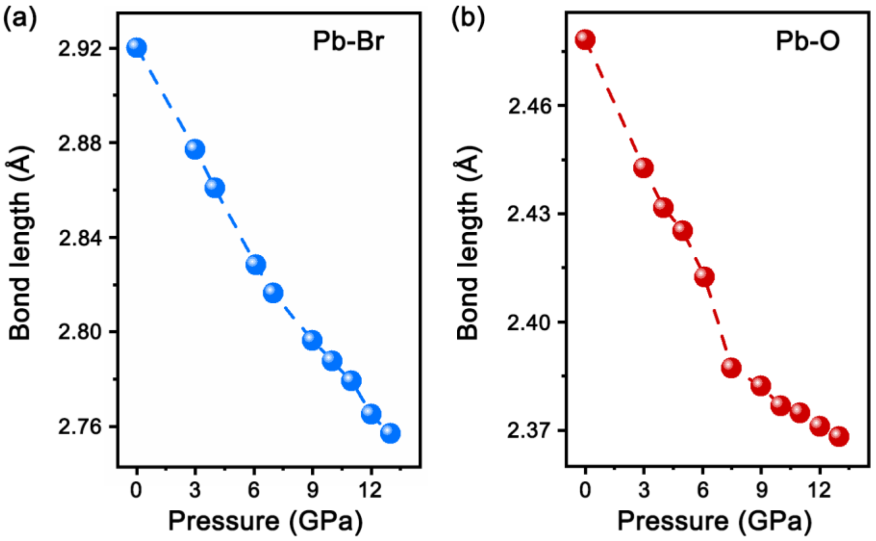

Figure S23. Evolution of bond lengths of (a) Pb-Br and (b) Pb-O as a function of pressure.

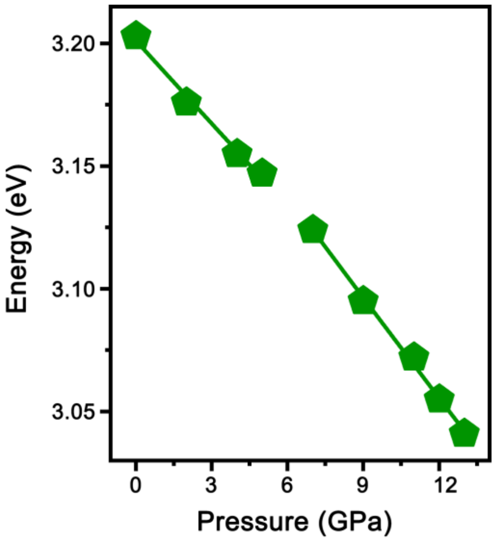

Figure S24. Evolution of the theoretical band gap values with increasing pressure.

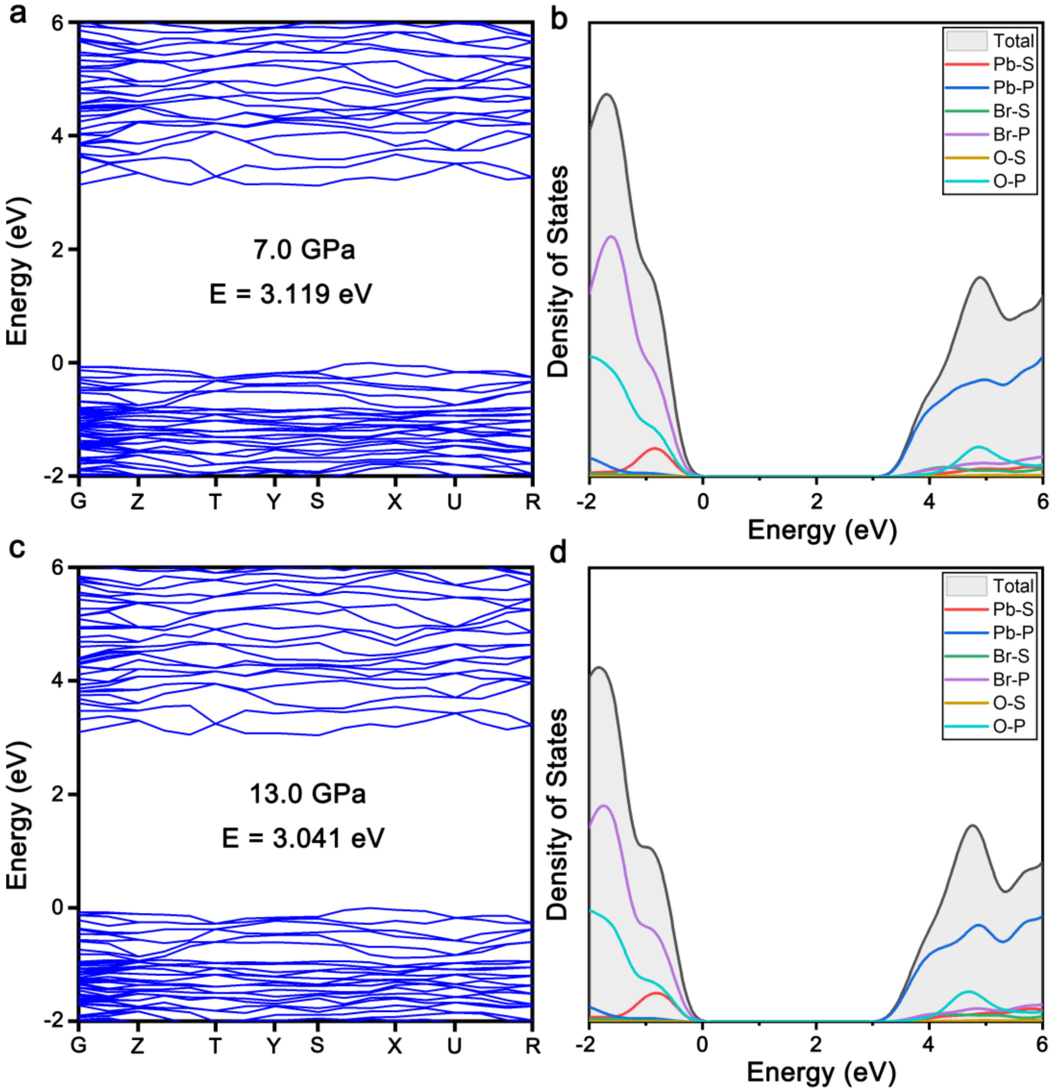

Figure S25. Calculated band structure and projected density of states of $[Pb_3Br_4][O_2C(CH_2)_2CO_2]$ at 7.0 GPa (a, b) and 13.0 GPa (c, d).

As an important factor affecting the optical properties of $[Pb_3Br_4][O_2C(CH_2)_2CO_2]$, it is important to investigate the change of band structure upon compression. Based on high-pressure absorption experiment, it indicated that the band gap narrowed continuously under pressure, and the decay rate increased after the isostructural phase transition. To elucidate the mechanism underlying of the reduction in band gap and the increase in decay rate, we choose Br-Pb-O unit for analysis (Figure S21-S23). Below 7.0 GPa, the Br-Pb-O bond angle was almost constant. The decrease of Pb-O and Pb-Br bond lengths, which promoted the overlap of elements orbitals, was the main reason for the narrow of band gap. Further compression, Pb-O and Pb-Br bond lengths further decreased and the Br-Pb-O bond angle started to shrink. The contraction of bond length and bond angle led to the intensification of the coupling degree between Pb 6 s, Br 4p and O 2p orbitals, and then the decay rate increased. On the basis of the refined structure, we further calculated the band structure and projected density of states of $[Pb_3Br_4][O_2C(CH_2)_2CO_2]$ at selected pressure. It can be observed from Figure S24 that the variation trend of the theoretical band gap was consistent with the experimental results. Further the projected density of states showed that the decrease of band gap was related to the increase of valence band maximum (VBM) attributable to the enhanced coupling between the 6s orbital of Pb, the 4p orbital of Br and the 2p orbital of O caused by the contraction of Br-Pb-O bond lengths and bond angle (Figure S25). The CBM was less sensitive to the shrinkage of structure due to the nonbonding characteristic of Pb 6p orbitals. [3]

References

[1] G. Xiao, Y. Cao, G. Qi, L. Wang, C. Liu, Z. Ma, X. Yang, Y. Sui, W. Zheng, B. Zou, *J. Am. Chem. Soc.* **2017**, *139,* 10087-10094.
[2] Y. Shi, W. Zhao, Z. Ma, G. Xiao, B. Zou, *Chem. Sci.* **2021**, *12*, 14711.

[3] X. Jing, D. Zhou, R. Sun, Y. Zhang, Y. Li, X. Li, Q. Li, H. Song, B. Liu, *Adv. Funct. Mater.* **2021**, *31*, 2100930.

[4] Y. Cao, G. Qi, L. Sui, Y. Shi, T. Geng, D. Zhang, K. Wang, K. Yuan, G. Wu, G. Xiao, S. Lu, B. Zou, *ACS Materials Lett.* **2020**, *2*, 381-388.

[5] X. Lou, L. Yao, N. Sui, Z. Kang, F. Li, Q. Zhou, Y. Li, M. Ni, H. Zhang, Y. Wang, *J. Phys. Chem. C* **2021**, *125*, 1041-1047.

[6] L. Kong, J. Gong, Q. Hu, F. Capitani, A. Celeste, T. Hattori, A. Sano-Furukawa, N. Li, W. Yang, G. Liu, H. Mao, *Adv. Funct. Mater.* **2021**, *31*, 2009131.

[7] T. Zheng, L. Luo, P. Du, S. Lis, U. R. Rodríguez-Mendoza, *Chem. Eng. J.* **2022**, *446*, 136839.

[8] L. Zhang, Z. Liu, X. Sun, G. Niu, J. Jiang, Y. Fang, D. Duan, K. Wang, L. Sui, K. Yuan, G. Wu, B. Zou, *Adv. Opt. Mater.* **2022**, *10*, 2101892.

[9] L. Zhang, Y. Fang, L. Sui, J. Yan, K. Wang, K. Yuan, W. Mao, B. Zou, *ACS Energy Lett.* **2019**, *4*, 2975-2982.

[10] Z. Ma, Q. Li, J. Luo, S. Li, L. Sui, D. Zhao, K. Yuan, G. Xiao, J. Tang, Z. Quan, B. Zou, *J. Am. Chem. Soc.* **2021**, *143*, 15176-15184.

[11] a) J. Yin, H. Yang and H. Fei, *Chem. Mater.* **2019**, *31*,3909-3916; b) L. Zhang, L. Wu, K. Wang, B. Zou, *Adv. Sci.* **2019**, *6*, 1801628.

[12] a) Y. Fang, J. Wang, L. Zhang, G. Niu, L. Sui, G. Wu, K. Yuan, K. Wang, B. Zou, *Chem. Sci.* **2023**, *14*, 2652-2658; b) S. Sajjadi, A. Khataee, N. Bagheri, M. Kobya, A. Şenocak, E. Demirbas, A. G. Karaoğlu, *J. Ind. Eng. Chem.* **2019**, *77*, 280-290.

[13] Z. Ma, F. Li, L. Sui, Y. Shi, R. Fu, K. Yuan, G. Xiao, B. Zou, *Adv. Opt. Mater.* **2020**, *8*, (18).
